# Association of *VDR* and *CYP2R1* Polymorphisms with Mite-Sensitized Persistent Allergic Rhinitis in a Chinese Population

**DOI:** 10.1371/journal.pone.0133162

**Published:** 2015-07-15

**Authors:** Hui-Qin Tian, Xin-Yuan Chen, Ying Lu, Wen-Min Lu, Mei-Lin Wang, Hai-Long Zhao, Mei-Ping Lu, Han Zhou, Ruo-Xi Chen, Zheng-Dong Zhang, Chong Shen, Lei Cheng

**Affiliations:** 1 Department of Otorhinolaryngology, The First Affiliated Hospital, Nanjing Medical University, Nanjing, China; 2 Department of Group Healthcare, The First Affiliated Hospital, Nanjing Medical University, Nanjing, China; 3 Department of Molecular and Genetic Toxicology, School of Public Health, Nanjing Medical University, Nanjing, China; 4 Department of Epidemiology and Biostatistics, School of Public Health, Nanjing Medical University, Nanjing, China; 5 International Centre for Allergy Research, Nanjing Medical University, Nanjing, China; Duke Cancer Institute, UNITED STATES

## Abstract

As recent studies have described an association between vitamin D and allergic rhinitis, we hypothesized that vitamin D pathway-related genes may be candidate genes for susceptibility to allergic rhinitis. Thus, we sought to evaluate whether polymorphisms in the vitamin D receptor (*VDR*) and *CYP2R1* genes are associated with mite-sensitized persistent allergic rhinitis (PER) in a Han Chinese population. A hospital-based case-control study consisting of 519 patients with mite-sensitized PER and 447 healthy controls was conducted. Five single nucleotide polymorphisms (SNPs) in *VDR* and *CYP2R1* were selected for genotyping. The genotype and allele frequencies of rs9729, rs2228570, rs1544410, and rs731236 in *VDR* as well as rs2060793 in *CYP2R1* were not significantly associated with susceptibility to mite-sensitized PER. After stratification analyses, however, both the *CT* and *CT/TT* genotypes of rs2228570 in *VDR* exhibited a significantly decreased risk (*CT*: adjusted odds ratio (OR)=0.58, 95% confidence intervals (CI)=0.37-0.91; *CT/TT*: adjusted OR=0.61, 95% CI=0.40-0.93) of mite-sensitized PER, while the *AA* genotype of rs2060793 in *CYP2R1* exhibited a significantly increased risk (adjusted OR=1.85, 95% CI=1.03-3.34) of PER in the age subgroup of <16 years old. Both the *AG* and *AG/GG* genotypes of rs731236 in *VDR* exhibited a significantly decreased risk (*AG*: adjusted OR=0.43, 95% CI=0.21-0.89; *AG/GG*: adjusted OR=0.46, 95% CI=0.23-0.94) of PER in the female subgroup. Analysis of the locus-locus interactions of *VDR* and *CYP2R1* revealed two models that involved combined SNPs of *VDR* and *CYP2R1* were statistically significant (*P*<0.05). Our data suggest that age and gender may have an impact on the association of three SNPs (rs2228570, rs731236, and rs2060793) in genes of the vitamin D pathway with the risk of mite-sensitized PER in this Chinese population. The *VDR* and *CYP2R1* variants may be involved in genetic interactions in the pathogenesis of PER.

## Introduction

Allergic rhinitis is a symptomatic disorder of the nose and is induced after allergen exposure by an immunoglobulin E (IgE)-mediated inflammation of the membranes lining the nose. Based on the frequency of symptoms, allergic rhinitis is classified as “intermittent” (IAR, < 4 days per week or < 4 weeks per year) or “persistent” (PER, > 4 days per week and > 4 weeks per year) [1,2]. Allergic rhinitis is a global health problem that causes major illness and disability worldwide, affecting the quality of life of affected individuals. Although clinical practice guidelines for allergic rhinitis have been developed over the past two decades and have resulted in improved care of patients [3], its exact pathogenesis remains unclear. Both environmental factors and genetic susceptibility are thought to play critical roles in the development of allergic rhinitis.

Recent studies have suggested that vitamin D may be associated with allergic rhinitis [4–6], although inconsistent results have implicated both vitamin D deficiency and supplementation. Vitamin D was originally considered an essential nutrient for the human body, but actually, this vitamin is a steroid hormone that is part of a complex endocrine pathway termed the “vitamin D endocrine system” [7]. Vitamin D has immunomodulatory effects on cells (T lymphocytes, B lymphocytes, and dendritic cells) and cytokines (e.g., interleukin (IL)-4, interferon (IFN)-γ, IL-12, IL-2, and IL-10) involved in the innate and adaptive immune responses [8]. Furthermore, vitamin D affects the Th1/Th2 balance by inhibiting Th1 cells and augmenting Th2 cell development [9]. Thus, based on these functions, vitamin D may play an important role in the pathogenesis of allergic rhinitis.

The activation and proper function of vitamin D require its nuclear receptor, vitamin D receptor (VDR), and several metabolizing enzymes such as vitamin D binding protein (VDBP), vitamin D-25-hydroxylase (25-OHase), and 25-hydroxyvitamin D-1-alpha-hydroxylase (1α-OHase). The *VDR* and *CYP2R1* genes encode VDR and microsomal 25-OHase, respectively [10], and the latter two are found in cells of the immune system [11,12]. In addition, the *VDR* and *CYP2R1* genes map to chromosome 12q and 11p respectively, and both of these chromosome locations include susceptibility loci for allergic diseases according to genome-wide linkage analysis studies [13]. Furthermore, both *VDR* and *CYP2R1* polymorphisms have been reported to be correlated with susceptibility to other allergic diseases, such as asthma [14,15]. Based on these previous findings, we hypothesize that *VDR* and *CYP2R1* are candidate genes for susceptibility to allergic rhinitis. The aim of this study was to investigate the contribution of single nucleotide polymorphisms (SNPs) of *VDR* and *CYP2R1* to the risk of PER due to house dust mites in a Han Chinese population.

## Results

### Clinical characteristics of the study subjects

The demographic characteristics and selected variables of the study population are shown in Table 1. The median age for the mite-sensitized PER patients was 16.0 (11.0–27.0) years (range 3–66 years), and this cohort included 345 males (66.5%) and 174 females (33.5%). The subjects in the control group had a median age of 15.0 (9.9–29.0) years (range 3–63 years) and included 277 males (62.0%) and 170 females (38.0%). No significant difference in the distribution of age (*P* = 0.453) or gender (*P* = 0.145) between the cases and controls was noted. Serum levels of total IgE (254.6 [117.0–552.0] kU/L) in PER patients were significantly higher (*P*<0.001) than those in healthy controls (25.3 [10.5–47.8] kU/L). In patients with PER, the serum levels of allergen-specific IgE against *Der p* and *Der f* were 19.9 (4.9–46.3) kU_A_/L and 17.2 (5.1–43.8) kU_A_/L, respectively.

**Table 1:** Distribution of selected variables between cases and controls.

Variables	Cases (n = 519)		Controls (*n* = 447)		*P*
	n	%	n	%	
Age, median (IQR)	16.0 (11.0–27.0)		15.0 (9.9–29.0)		0.453
Gender					
Male	345	66.5	277	62.0	0.145
Female	174	33.5	170	38.0	
Concomitant asthma ^a^					
Yes	118	27.0			
No	319	73.0			
Serum total IgE (kU/L), median (IQR)	254.6 (117.0–552.0)		25.3 (10.5–47.8)		<0.001
Allergen-specific IgE (kU_A_/L), median (IQR)					
*Dermatophagoides pteronyssinus*	19.9 (4.9–46.3)				
*Dermatophagoides farinae*	17.2 (5.1–43.8)				

^a^ Information of concomitant asthma was unavailable in some cases.

IQR: interquartile range.

### SNPs of *VDR* and *CYP2R1*

The primary information and allele frequencies for the five selected SNPs in *VDR* and *CYP2R1* genes are summarized (Table 2). All genotype distributions of the control group were consistent with those expected based on the Hardy-Weinberg equilibrium (HWE; *P*>0.05). In addition, the minor allele frequency (MAF) for each of the five SNPs was consistent with that reported in the NCBI database (http://www.ncbi.nlm.nih.gov/SNP/).

**Table 2:** Primary information about genotyped SNPs in *VDR* and *CYP2R1*.

SNPs	Location	Base change	MAF			*P* for HWE^b^	Genotyped (%)
			NCBI^a^	Case	Control		
***VDR***							
rs9729	3’UTR	*C*>*A*	0.317	0.279	0.272	0.806	99.9
rs2228570	Exon 2	*C*>*T*	0.366	0.463	0.490	0.806	99.8
rs1544410	Intron 8	*G*>*A*	0.061	0.048	0.049	0.282	99.4
rs731236	Exon 9	*A*>*G*	0.061	0.050	0.053	0.816	99.5
***CYP2R1***							
rs2060793	Promoter	*G*>*A*	0.354	0.379	0.349	0.659	99.7

^a^ MAF from the NCBI database (http://www.ncbi.nlm.nih.gov/SNP/).

^b^ *P*-value compared in the control group to that expected by HWE.

SNPs, single nucleotide polymorphisms; *VDR*, vitamin D (1,25-dihydroxyvitamin D3) receptor (*Homo* sapiens [human]); *CYP2R1*, cytochrome P450, family 2, subfamily R, polypeptide 1 (*Homo* sapiens [human]); MAF, minor allele frequency; HWE, Hardy-Weinberg equilibrium; UTR, untranslated region.

### Association between individual SNPs and PER

The results of genotypic and allele frequency analysis of the five selected SNPs from cases and controls are shown in Table 3. The genotypes of rs9729, rs2228570, rs1544410, and rs731236 in *VDR* as well as rs2060793 in *CYP2R1* were not significantly associated with susceptibility to mite-sensitized PER, although a tendency for increased risk neared our predetermined significance level (adjusted OR = 1.16, 95% CI = 0.96–1.39) for the *A* allele of rs2060793 in *CYP2R1*.

**Table 3:** Genotype and allele frequencies of *VDR* and *CRY2R1* polymorphisms among cases and controls.

Genotypes	Cases		Controls		Crude OR (95% CI)	Adjusted OR (95% CI) ^b^ ^,^ ^c^
	n	%	n	%		
***VDR***						
**rs9729**	*n* = 518		*n* = 447			
*CC*	266	51.4	236	52.8	1.00	1.00
*CA*	215	41.5	179	40.0	1.07 (0.82–1.39)	1.07 (0.82–1.39)
*AA*	37	7.1	32	7.2	1.03 (0.62–1.70)	1.02 (0.62–1.70)
*CA*/*AA*	252	48.6	211	47.2	1.06 (0.82–1.37)	1.06 (0.82–1.37)
C allele ^a^					1.04 (0.85–1.27)	1.04 (0.85–1.23)
**rs2228570**	*n* = 517		*n* = 447			
*CC*	147	28.4	115	25.7	1.00	1.00
*CT*	261	50.5	226	50.6	0.90 (0.67–1.22)	0.91 (0.67–1.23)
*TT*	109	21.1	106	23.7	0.80 (0.56–1.15)	0.81 (0.56–1.16)
*CT*/*TT*	370	71.6	332	74.3	0.87 (0.66–1.16)	0.88 (0.66–1.17)
*T* allele ^a^					0.90 (0.75–1.08)	0.90 (0.75–1.08)
**rs1544410**	*n* = 518		*n* = 442			
*GG*	470	90.7	399	90.3	1.00	1.00
*GA*	46	8.9	43	9.7	0.91 (0.59–1.41)	0.90 (0.58–1.39)
*AA*	2	0.4	0	0.0	—	—
*GA*/*AA*	48	9.3	43	9.7	0.95 (0.62–1.46)	0.94 (0.61–1.45)
*A* allele ^a^					0.99 (0.65–1.51)	0.98 (0.64–1.49)
**rs731236**	*n* = 518		*n* = 443			
*AA*	469	90.5	397	89.6	1.00	1.00
*AG*	46	8.9	45	10.2	0.87 (0.56–1.33)	0.86 (0.56–1.33)
*GG*	3	0.6	1	0.2	2.54 (0.26–24.5)	2.49 (0.26–24.1)
*AG*/*GG*	9	9.5	46	10.4	0.90 (0.59–1.38)	0.90 (0.59–1.37)
*G* allele ^a^					0.94 (0.63–1.41)	0.94 (0.63–1.41)
***CYP2R1***						
**rs2060793**	*n* = 519		*n* = 444			
*GG*	201	38.7	186	41.9	1.00	1.00
*GA*	243	46.8	206	46.4	1.11 (0.83–1.43)	1.11 (0.84–1.46)
*AA*	75	14.5	52	11.7	1.34 (0.89–2.00)	1.38 (0.92–2.08)
*GA*/*AA*	318	61.3	258	58.1	1.14 (0.88–1.48)	1.16 (0.90–1.51)
*A* allele ^a^					1.14 (0.94–1.37)	1.16 (0.96–1.39)

^a^ Additive model.

^b^ Adjusted for age and gender in logistic regression model.

^c^ Thousand times permutation test.

*VDR*, vitamin D (1,25-dihydroxyvitamin D3) receptor (*Homo* sapiens [human]); *CYP2R1*, cytochrome P450, family 2, subfamily R, polypeptide 1 (*Homo* sapiens [human]); OR, odds ratio; CI, confidence interval.

### Stratification analysis of PER subgroups

In the stratification analysis, PER patients were divided into four subgroups by age, gender, presence of asthma, and total IgE levels [16]. As shown in Table 4, compared with wild-type *GG* genotype, the homozygote *AA* genotype of rs2060793 in *CYP2R1* exhibited a significantly increased risk of mite-sensitized PER (adjusted OR = 1.85, 95% CI = 1.03–3.34) in the age subgroup of <16 years old, and the significance remained after a thousand times permutation test. No significant associations were found between the genotypes of rs2060793 in *CYP2R1* and the other PER subgroups. As for the four SNPs in *VDR*, compared with their wild-type genotypes, both the *CT* and *CT/TT* genotypes of rs2228570 exhibited a significantly decreased risk of mite-sensitized PER (*CT*: adjusted OR = 0.58, 95% CI = 0.37–0.91; *CT/TT*: adjusted OR = 0.61, 95% CI = 0.40–0.93) in the age subgroup of <16 years old. In addition, both the *AG* and *AG/GG* genotypes of rs731236 exhibited a significantly decreased risk of mite-sensitized PER (*AG*: adjusted OR = 0.43, 95% CI = 0.21–0.89; *AG/GG*: adjusted OR = 0.46, 95% CI = 0.23–0.94) in the female subgroup, and this significance remained after analysis with a thousand times permutation test. No significant associations were found between the genotypes of rs2228570 and rs731236 in *VDR* and the other PER subgroups. In addition, no significant associations were detected between the genotypes of rs9729 and rs1544410 in *VDR* and the PER subgroups (*P*>0.05, data not shown).

**Table 4:** Stratification analyses of *VDR* and *CRY2R1* polymorphisms in PER subgroups.

SNPs	Variables	Subgroup	N (cases/controls) ^b^	Adjusted OR (95% CI) ^c^ ^,^ ^d^		
				XY ^e^	YY ^e^	XY/YY ^e^
***VDR***						
**rs2228570**						
	Age (years)	<16	249/224	0.58(0.37–0.91)	0.68(0.40–1.15)	0.61(0.40–0.93)
		≥16	268/223	1.38(0.91–2.10)	0.94(0.57–1.54)	1.22(0.82–1.80)
	Gender	Male	345/277	0.85(0.58–1.25)	0.81(0.51–1.26)	0.84(0.59–1.20)
		Female	172/170	0.97(0.59–1.62)	0.80(0.43–1.49)	0.92(0.57–1.49)
	Concomitant asthma	Yes	118/447	1.06(0.63–1.77)	1.03(0.56–1.90)	1.05(0.64–1.72)
		No	318/447	0.91(0.64–1.27)	0.76(0.50–1.15)	0.86(0.62–1.19)
	Total IgE level ^a^	Lower	456/447	0.87(0.64–1.19)	0.81(0.56–1.17)	0.85(0.63–1.14)
		Higher	51/447	1.14(0.56–2.34)	0.78(0.31–1.93)	1.03(0.52–2.05)
**rs731236**						
	Age (years)	<16	248/224	1.20(0.62–2.34)	0.81(0.05–13.06)	1.18(0.62–2.26)
		≥16	270/219	0.67(0.38–1.20)	—	0.73(0.41–1.28)
	Gender	Male	344/275	1.37(0.76–2.45)	1.62(0.15–18.13)	1.38(0.78–2.43)
		Female	174/168	0.43(0.21–0.89)	—	0.46(0.23–0.94)
	Concomitant asthma	Yes	117/443	0.82(0.38–1.76)	2.53(0.16–41.08)	0.88(0.42–1.83)
		No	319/443	0.93(0.57–1.52)	1.33(0.08–21.40)	0.94(0.58–1.53)
	Total IgE level ^a^	Lower	457/443	0.87(0.55–1.36)	1.89(0.17–21.03)	0.89(0.57–1.38)
		Higher	51/443	0.82(0.28–2.40)	9.92(0.57–172.91)	1.01(0.38–2.68)
***CYP2R1***						
**rs2060793**						
	Age (years)	<16	249/224	1.30(0.88–1.92)	1.85(1.03–3.34)	1.40(0.97–2.03)
		≥16	270/220	0.92(0.64–1.35)	0.98(0.55–1.72)	0.93(0.64–1.34)
	Gender	Male	345/274	1.07(0.76–1.50)	1.37(0.80–2.33)	1.12(0.81–1.55)
		Female	174/170	1.20(0.75–1.91)	1.35(0.71–2.56)	1.24(0.80–1.91)
	Concomitant asthma	Yes	118/444	1.05(0.67–1.64)	1.33(0.69–2.58)	1.11(0.72–1.68)
		No	319/444	1.22(0.90–1.67)	1.45(0.91–2.30)	1.27(0.94–1.71)
	Total IgE level ^a^	Lower	458/444	1.11(0.84–1.47)	1.35(0.89–2.05)	1.15(0.88–1.51)
		Higher	51/444	1.34(0.69–2.58)	2.26(0.97–5.28)	1.52(0.82–2.81)

^a^ Lower: below the 90th percentile of logarithmic total IgE level; Higher: above the 90th percentile of logarithmic total IgE level.

^b^ Controls were stratified accordingly when dividing cases into age and gender subgroups, but they were kept as a whole in the situation of the other two subgroups, since the controls in the study had neither asthma nor higher total IgE level in the first place (see Table 1).

^c^ Adjusted for age and gender in logistic regression model.

^d^ Thousand times permutation test.

^e^ XY: heterozygote genotype/wild-type genotype; YY: homozygote genotype/wild-type genotype; XY/YY: heterozygote genotype +homozygote genotype/wild-type genotype.

PER, persistent allergic rhinitis; *VDR*, vitamin D (1,25-dihydroxyvitamin D3) receptor (*Homo* sapiens [human]); *CYP2R1*, cytochrome P450, family 2, subfamily R, polypeptide 1 (*Homo* sapiens [human]); OR, odds ratio; CI, confidence interval.

### Locus-locus interactions of *VDR* and *CYP2R1*

The genotypes of rs9729, rs2228570, rs1544410, rs731236, and rs2060793 were renamed as A1, A2, A3, A4, and A5, respectively. The multifactor dimensionality reduction (MDR) software was used to consider the joint effects of the five SNPs in *VDR* and *CYP2R1*. As presented in Table 5, two models had statistical significance (*P*<0.05). The cross-validation consistency of model A1 A2 A5 and model A1 A2 A4 A5 was 10/10 for each. Model A1 A2 A4 A5 was considered the best model due to the highest test balanced accuracy (0.5653) of the models tested.

**Table 5:** Multifactor dimensionality reduction models for locus-locus interactions.

Model ^a^	Training balance accuracy	Test balance accuracy	Cross-validation consistency	*P*
A5	0.5180	0.4938	8/10	0.6230
A2 A5	0.5451	0.5199	10/10	0.3770
A1 A2 A5	0.5788	0.5611	10/10	0.0107
A1 A2 A4 A5	0.5926	0.5653	10/10	0.0107
A1 A2 A3 A4 A5	0.5988	0.5539	10/10	0.1719

^a^ The genotypes of rs9729, rs2228570, rs1544410, rs731236, and rs2060793 were renamed as A1, A2, A3, A4, and A5, respectively.

## Discussion

As a multifactorial disease, allergic rhinitis involves a genetic component; however, the identification of susceptibility genes is not straightforward. Various molecules participate in the phases of an allergic reaction, and as such, many genes encoding those molecules can be candidate genes for allergic rhinitis. Previous reports revealed that several genetic variants of candidate genes, such as *ADAM33* [16,17], *IL4* [18,19], *MRPL4*, and *TNFA* [20], are positively associated with susceptibility to allergic rhinitis.

As mentioned before, vitamin D has known immunomodulatory effects and has been suggested to play a role in the pathogenesis of allergic rhinitis. Therefore, genes encoding players in the vitamin D pathway are potentially candidate genes involved in the genetic etiology of allergic rhinitis. The *VDR* gene is located on chromosome 12q13.11 and is comprised of 11 exons that, together with intervening introns, span approximately 75 kb [21]. The *CYP2R1* (cytochrome P450, family 2, subfamily R, polypeptide 1) gene is located on chromosome 11p15.2 and contains 18620 bp (*see* http://www.ncbi.nlm.nih.gov/gene/120227). The relationship between vitamin D and allergic diseases has increasingly attracted attention in recent years, and accordingly, SNPs of *VDR* and *CYP2R1* have also been reported to be associated with some immune disorders [22–27]. The contribution of these SNPs to the susceptibility to allergic rhinitis, however, has not yet been reported.

To clarify the association between the variants of these two candidate genes involved in the vitamin D endocrine system and the risk of mite-sensitized PER in the Han Chinese population, we conducted a case-control study to analyze five selected SNPs in *VDR* and *CYP2R1*. To the best of our knowledge, this is the first study reported to evaluate the relationship between *VDR* and *CYP2R1* genetic variations and allergic rhinitis.

The five SNPs that are either functional or frequently studied in the context of other immune diseases [22–27]. The rs9729 is located in the *VDR* 3’ UTR, which regulates mRNA stability, thereby modulating VDR expression. Studies of the rs9729 polymorphism are rare, but one paper [22] reported that a haplotype contained rs9729 in a German population was negatively associated with type 1 diabetes, an autoimmune disease that results in T cell-mediated destruction of insulin-producing cells within the pancreas. The rs2228570, rs1544410, and rs731236 polymorphisms in *VDR* are relatively well studied compared to rs9729. These SNPs are also referred to as *FokI*, *BsmI*, and *TaqI* respectively, due to the corresponding restriction enzymes used in restriction fragment length polymorphism (RFLP) analysis. A *T* to *C* polymorphism of *FokI* results in two protein variants: a long version of the VDR protein (*f* allele) and a protein shortened by three amino acids (*F* allele) [28]. van Etten *et al* [29] inferred in their work that *VDR FokI* polymorphism affects immune cell behavior, with a more active immune system for the short *F*-VDR. Thus, this polymorphism may play a role in immune-mediated diseases. *BsmI* resides in the region of intron 8, near the 3’UTR of *VDR*. *TaqI*, which is also near the 3’UTR, is within exon 9; however, the *A*/*G* polymorphism yields synonymous coding sequence. The effects of *BsmI* and *TaqI* polymorphisms are either vague or negative, but these SNPs may be in linkage disequilibrium with other truly functional polymorphisms located elsewhere. Mostowska *et al* [23] studied these three SNPs in systemic lupus erythematosus (SLE) patients and healthy individuals in a Polish population and reported no significant difference between these two groups, although a significant association was found between the *F*/*F* and *F*/*f* allele with renal manifestations of SLE (OR = 3.228 [1.534–6.792]). Hitchon *et al* [24] reported that the *FokI* SNP was significantly associated with rheumatoid arthritis in North American natives. Recently, Maalmi *et al* [25] found the polymorphisms of *FokI*, *BsmI*, and *TaqI* were significantly associated with asthma in Tunisian children; however, a study from Li *et al* [26] suggested that *FokI* and *BsmI* polymorphisms of *VDR* may not significantly contribute to the development of asthma in the Chinese Hans population. On the other hand, the rs2060793 is in the *CYP2R1* neargene-5 region, which is the promoter region and is known to be associated with the regulation of gene transcription [30]. The relationship between the SNPs of *CYP2R1* and susceptibility to allergic diseases has not been fully explored. We found only one study by Wang *et al* [27] that reported that the polymorphism of rs2060793 was a risk factor for increased peripheral eosinophil levels in Chinese children with eczema.

Despite the above reports of associations between *VDR* and *CYP2R1* polymorphisms and immune diseases, the relationship between genetic variations in the vitamin D pathway-related genes and PER susceptibility has not been described. In this study, we found a lack of association between the *VDR* and *CYP2R1* polymorphisms and the risk of mite-sensitized PER in a Han Chinese population; however, upon stratification of the PER group, we found that compared with their wild-type genotypes, the *CT* and *CT/TT* genotypes of rs2228570 in *VDR* exhibited a significantly decreased risk in the age subgroup of <16 years old, while the *AA* genotype of rs2060793 in *CYP2R1* exhibited a significantly increased risk of mite-sensitized PER in the age subgroup of <16 years old. Moreover, both the *AG* and *AG/GG* genotypes of *VDR* rs731236 exhibited a significantly decreased risk of PER in the female subgroup. Therefore, PER patients of <16 years old and PER females may be susceptible to the genetic effect caused by *VDR* and *CYP2R1*. Interestingly, Mai *et al* [6] recently reported that vitamin D appears to play different roles in the development of allergic rhinitis among men and women in Norway and that lower serum 25(OH)D levels were associated with an increased risk of allergic rhinitis among men but a reduced risk of allergic rhinitis among women. Meanwhile, other conflicting results have been described about the relationship between vitamin D levels and the risk of allergic rhinitis in adults and children [4,5]. Our results offer genetic insight that complements these epidemiological studies. As for subgroups of presence of asthma and total IgE levels, based on the results that no significant differences were found between *VDR* or *CYP2R1* genotypes with all the relevant PER subgroups, it is suggested that asthma and total IgE levels have no influence upon the relationship between *VDR* or *CYP2R1* genotypes and the risk of mite-sensitized PER in our study. Those results are possible, because the relationship between *VDR* or *CYP2R1* genotypes and the risk of asthma has not been determined with inconsistent results in different ethnic groups [25,26], what’s more, total IgE level is not a specific index for the diagnosis of PER. Thus, further studies are necessary to more adequately explore the association between polymorphisms in *VDR*, *CYP2R1*, and even other vitamin D pathway-related genes and PER susceptibility on a larger scale and also in different ethnic regions.

The MDR method following conventional statistical analyses were used in our study in order to consider the joint effects of five SNPs in the *VDR* and *CYP2R1* genes. We found that two models (A1A2A5 and A1A2A4A5) were statistically significant, and the model of A1A2A4A5 (representing rs9729, rs2228570, rs731236, and rs2060793) was the best model. From this model, three SNPs (rs2228570, rs731236, and rs2060793) were found to be significantly associated with different PER subgroups in this study. Since PER is a polygenic and multifactorial disease, our results suggest that the multigenic interactions among loci in different genes of the vitamin D endocrine system may occur in the pathogenesis of PER.

Finally, several limitations of our present study should be addressed. First, our study was a case-control study in a hospital-based setting, and as a result, these subjects may not be representative of the general population. Secondly, we did not detect VDR protein levels or serum vitamin D levels in our study subjects, which may have validated the differences we found at the gene level. Serum vitamin D levels in the human body are influenced by multiple factors, such as exposure to sunshine, diet, body mass index, accompanying diseases, and skin color. In addition, VDR protein is expressed in different types of immune cells but not in nasal tissue [11]. As a result, subsequent validation studies should be designed to evaluate these issues. The third limitation of our study was that other vitamin D-related genes were not examined in the present study, and study of additional genes will be useful for complementing these studies.

In conclusion, our findings suggest that age and gender may have an impact on the association of three SNPs (rs2228570, rs731236, and rs2060793) in genes of the vitamin D pathway with the risk of mite-sensitized PER in this Chinese population. The *VDR* and *CYP2R1* variants may be involved in genetic interactions in the pathogenesis of PER.

## Materials and Methods

### Study subjects

All subjects were unrelated Chinese Hans from the Jiangsu and Anhui provinces in eastern China. We employed a hospital-based case-control design in our study. A total of 519 patients with mite-sensitized PER and 447 healthy controls were recruited. All patients were enrolled in the Department of Otorhinolaryngology and Allergy Clinic at the First Affiliated Hospital of Nanjing Medical University (Nanjing, China) between May 2008 and December 2013, and the diagnosis of PER was based on the guidelines of the “Allergic Rhinitis and its Impact on Asthma” (ARIA) 2008 update [2]. The recruited controls were from the annual physical examinations and exhibited neither clinical manifestation nor family history of rhinitis, asthma, or atopic diseases. The control individuals were frequency matched to the cases by age (±5 years) and gender. A standard questionnaire was administered through face-to-face interviews by trained interviewers to collect demographic data and related information. The response rate for this study was 95% in the case group and 90% in the control group. After the interview, 5 mL of peripheral blood was collected from each subject. All data and sample collections for this study were approved by the ethics committee of Nanjing Medical University, and written informed consent was obtained from all participants or the next of kin, caretakers, or guardians on behalf of the minors/children enrolled in our study. All the implements were under full compliance with all government policies and the Helsinki declaration.

### Allergy testing

Serum total IgE and specific IgE levels were determined using ImmunoCAP assays (Phadia, Uppsala, Sweden). Total IgE levels were measured both in the PER group and the control group. Specific IgE antibodies to common aeroallergens, including *Dermatophagoides pteronyssinus* (*Der p*; d1), *Dermatophagoides farinae* (*Der f*; d2), cat dander (e1), dog dander (e5), *Blatella germanica* (i6), *Alternaria alternate* (m6), *Ambrosia artemisiifolia* (w1), and *Artemisia vulgaris* (w6), were determined in PER group. Specific IgE concentrations of ≥0.35kU_A_/L were defined as positive. All patients were sensitized to house dust mites (*Der p* and/or *Der f*), and 134 of these cases (25.8%) were accompanied by sensitization to other aeroallergens.

### SNP selection

Based on the HapMap data (http://hapmap.ncbi.nlm.nih.gov/) and dbSNP data (http://www.ncbi.nlm.nih.gov/SNP/), SNPs in the *VDR* and *CYP2R1* genes were chosen as follows: 1) location in the 5′ flanking regions, 5′ UTR, 3′ UTR, and coding regions with amino acid changes or frequently studied variants in previous reports and 2) MAF>5% in the Chinese population. Overall eight SNPs fulfilled the criteria: rs9729, rs2228570, rs7975232, rs1544410, rs731236, rs11574125, rs10741657, and rs2060793. Since rs9729 and rs7975232, as well as rs10741657 and rs2060793 are in complete linkage, respectively, we choose the functional rs9729, which is located in the 3′ UTR rather than rs7975232, which is in the intron region. We also randomly choose rs2060793 to genotype as both rs10741657 and rs2060793 are in the neargene-5 region. We did not analyze rs11574125 because the corresponding genotyping probe failed. This is an insertion/deletion polymorphism and is also influenced by a nearby SNP. In the end, a total of five SNPs were analyzed: rs9729, rs2228570, rs1544410, and rs731236 in *VDR* and rs2060793 in *CYP2R1*.

### Genotyping

Genomic DNA was isolated from peripheral blood leukocytes by proteinase K digestion and phenol/chloroform extraction. Genotyping of the polymorphisms was performed with the TaqMan SNP Genotyping Assay in a 384-well ABI 7900HT Real-Time PCR System (Applied Biosystems, Foster City, CA, USA). SDS 2.3 software was used for allelic discrimination (Applied Biosystems). PCR primers and MGB TaqMan probes are listed in Table 6. The amplification was performed under the following conditions: 50°C for 2 min, 95°C for 10 min, and then 40–45 cycles of 95°C for 15 sec and 60°C for 1 min. About 10% of the samples were randomly selected for repeated assays for confirmation, and the results were 100% concordant. The genotyping success rates for the five SNPs were >99%.

**Table 6:** Primers and probes for genotype screening by TaqMan allelic discrimination.

SNPs ^a^	Primers	Probes
***VDR***		
rs9729	F: 5'- CAGTGGGAGAAAACACTTGTAAGTTG-3'	C: FAM-ATCCCCTCATT**C**AGGA-MGB
	R: 5'- CACGCCCTCCTCTGTCAGTT-3'	A: HEX-AATCCCCTCATT**A**AGGA-MGB
rs2228570	F: 5'-ACCGTGGCCTGCTTGCT-3'	C: FAM-CAGGGA**C**GGAGGC-MGB
	R: 5'-AGGGTCAGGCAGGGAAGTG-3'	T: HEX-ACAGGGA**T**GGAGGCA-MGB
rs1544410	F: 5'-GGGATTCTGAGGAACTAGATAAGCA-3'	G: FAM-CAGGCCTGC**G**CAT-MGB
	R: 5'-CCCGCAAGAAACCTCAAATAAC-3'	A: HEX-AGACAGGCCTGC**A**CA-MGB
rs731236	F: 5'-CCGTGCCCACAGATCGTC-3'	A: FAM- TGGCCTC**A**ATCAG-MGB
	R: 5'-GCGGATGTACGTCTGCAGTG-3'	G: HEX-ATGGCCTC**G**ATCAG-MGB
***CYP2R1***		
rs2060793	F: 5'-TCTTCCACTTATAAGGATCGTTGTGA-3'	G: FAM-CTGGATAATCCC**G**ACTC-MGB
	R: 5'-GCAGATGGAATTAAAGGGCTAATC-3'	A: HEX-CTGGATAATCCC**A**ACTC-MGB

^a^ SNP position in NCBI dbSNP (http://www.ncbi.nlm.nih.gov/SNP/).

SNPs, single nucleotide polymorphisms; *VDR*, vitamin D (1,25-dihydroxyvitamin D3) receptor (*Homo* sapiens [human]); *CYP2R1*, cytochrome P450, family 2, subfamily R, polypeptide 1 (*Homo* sapiens [human]).

### Statistical analysis

Allele frequencies were tested against departure from the HWE using a goodness-of-fit χ^2^ test before analysis. Differences in the distributions of demographic characteristics, selected variables, and frequencies of *VDR* and *CYP2R1* genotypes and alleles between PER patients and the control subjects were evaluated using the χ^2^ test (for categorical variables), Student’s *t*-test (for continuous variables), or nonparametric test. ORs and the corresponding 95% CIs were determined using unconditional logistic regression method with adjustments for possible confounders to estimate the association between *VDR* and *CYP2R1* polymorphisms and the risk of PER. All of the above statistical analyses were performed with Statistical Analysis System software 9.1.3 (SAS Institute, Cary, NC, USA). Additionally, to explore the potential interactions between the SNPs of *VDR* and *CYP2R1*, MDR software was used (version 3.0, available at the website http://www.epistasis.org). *P*-values smaller than 0.05 were considered statistically significant.
